# SKP2 High Expression, KIT Exon 11 Deletions, and Gastrointestinal Bleeding as Predictors of Poor Prognosis in Primary Gastrointestinal Stromal Tumors

**DOI:** 10.1371/journal.pone.0062951

**Published:** 2013-05-17

**Authors:** Ang Lv, Zhongwu Li, Xiuyun Tian, Xiaoya Guan, Min Zhao, Bin Dong, Chunyi Hao

**Affiliations:** 1 Key Laboratory of Carcinogenesis and Translational Research (Ministry of Education), Department of Hepato-Pancreato-Biliary Surgery, Peking University School of Oncology, Beijing Cancer Hospital & Institute, Beijing, People’s Republic of China; 2 Key Laboratory of Carcinogenesis and Translational Research (Ministry of Education), Department of Pathology, Peking University School of Oncology, Beijing Cancer Hospital & Institute, Beijing, People’s Republic of China; 3 Key Laboratory of Carcinogenesis and Translational Research (Ministry of Education), Center laboratory, Peking University School of Oncology, Beijing Cancer Hospital & Institute, Beijing, People’s Republic of China; Sapporo Medical University, Japan

## Abstract

**Background and Aims:**

Considering the indication of adjuvant therapy, the recurrence risk for primary gastrointestinal stromal tumor (GIST) after surgery needs to be accurately estimated. However, current risk stratification schemes may still have room for improvement. This study seeks to analyze prognostic factors for primary GISTs from 3 aspects, including clinicopathological parameters, immunohistochemical biomarkers, and gene mutational status, and attempts to find novel valuable factors predicting the malignancy potential of GISTs.

**Methods:**

Retrospective data from 114 primary GIST patients after R0 resection were collected. Clinicopathological data was obtained from medical records and re-evaluated. Immunohistochemical analysis was performed using the Tissue Microarray method for Ki67, p16, p27, p53, SKP2, CD133, and actin. KIT gene exons 9, 11, 13, and 17 and PDGFRα gene exons 12 and 18 were tested for mutations using PCR.

**Results:**

Univariate analysis revealed the following factors as poor prognostic indicators for relapse-free survival with a median follow-up of 50 months: male gender, gastrointestinal bleeding, mitotic index >5/50HPFs, tumor size >5 cm, non-gastric site, necrosis, epithelioid or mixed cell type, surrounding tissue invasion, Ki67>5%, p16>20%, p53 index >10, SKP2>10%, and KIT exon 11 deletion. Besides mitotic index, tumor size and site, SKP2 high expression (RR = 2.91, 95% CI: 1.41–5.99, *P = *0.004) and KIT exon 11 deletion (RR = 2.73, 95% CI: 1.04–7.16, *P = *0.041) were also independent risk factors in multivariate analysis, with gastrointestinal bleeding also showing a trend towards significance (RR = 1.88, 95% CI: 0.98–3.64, *P = *0.059). In addition, gastrointestinal bleeding and SKP2 high expression showed a good ability to stratify high-risk patients further.

**Conclusion:**

Our results show that gastrointestinal bleeding, SKP2 high expression, and KIT exon 11 deletions may be useful indicators of high recurrence risk for primary GIST patients.

## Introduction

Gastrointestinal stromal tumors (GISTs) are the most common mesenchymal neoplasms of the gastrointestinal tract with an annual incidence of 10–15 cases per million [1], [2].

The gold standard for treatment of localized primary GIST is surgical resection, although tumor recurrence or metastasis is common, particularly in the liver and peritoneum [3]. The introduction of imatinib mesylate (IM), a model of target-based molecular therapy, improves GIST prognosis dramatically [4]. However, the potential toxic effects and financial cost of this treatment are considerable. Therefore, predicting the recurrence risk and malignancy potential of GISTs more precisely remain valuable issues worth exploring.

GISTs have a wide spectrum of biological behaviors with varying malignancy potential, making it difficult to precisely distinguish between benign and malignant lesions. Mitotic index, tumor size, and tumor site, which can be important prognostic predictors for GIST patients, form the basis of a few risk-stratification schemes that have been developed for operable GIST, including NIH-Fletcher criteria, AFIP-Miettinen criteria (Table S1), and revised NIH consensus criteria [5]–[7]. Within the same risk category, however, the clinical behavior of GIST still vary, and thus, current risk stratification schemes may still have room for improvement [8].

Although several other clinicopathological factors have been associated with GISTs, none have been conclusively linked to GIST as prognostic markers or included in any risk stratification scheme. For example, tumor necrosis, predominant cell type, cellularity, and surrounding tissue invasion have all been previously reported as potential valuable prognostic factors for GIST, although no firm conclusions have been drawn [9]–[11]. Conversely, gastrointestinal bleeding is one of the most common symptoms of GIST patients and a sign of mucosa invasion, but it is seldom mentioned as a prognostic factor.

Over the last decade, an increasing number of studies have revealed a correlation between the clinical behavior of GIST and the expression of immunohistochemical biomarkers. More specifically, Ki67, a nuclear protein associated with cellular proliferation, is one of the most frequent prognostic markers investigated in the literature on GISTs. Further, some key cell-cycle regulatory proteins (CCRPs) including p53, p16, and p27 also represent hot spots in GIST research. Moreover, SKP2, which plays a role in soft-tissue sarcoma aggressiveness, CD133, and actin have all recently been reported as potential markers of GIST prognosis [10], [12], [13]. However, none of above mentioned biomarkers have been conclusively established or included in any risk stratification scheme. In this study, we attempted to examine a majority of the above mentioned prospective but unproven biomarkers as potential predictors for the prognosis of GIST.

Apart from these potential markers, activating mutations of KIT and, less commonly, of PDGFRα–which encode the stem-cell factor receptor (KIT) and platelet-derived growth factor receptor α (PDGFRα) tyrosine kinases, respectively–are believed to be critical in tumorigenesis in GIST 14,15. However, the importance of KIT and PDGFRα mutational status as a prognostic factor also remains controversial. In some previous studies, deletion of KIT exon 11 indicated a poor prognosis [16], whereas tumors with point mutations or duplications had a better prognosis [17]. Moreover, mutations in KIT exon 9 and PDGFRα exon 18 predict high and low malignant potential, respectively [18], [19].

Here, we analyzed the influence of these 3 different types of factors, including clinicopathological factors, immunohistochemical biomarkers, and gene mutational status, on the recurrence risk of R0 resected primary GISTs to identify potential new factors that may better predict their clinical behavior and prognosis.

## Materials and Methods

### Ethics Statement

For the use of clinical materials for research purposes, approval from the Regional Ethical Committees, Beijing Cancer Hospital, China was obtained. Written informed consents have been obtained from all participants; for subjects who were children, written informed consents have been obtained from their guardians. The clinical investigation was conducted according to the principles expressed in the Declaration of Helsinki.

### Patients

A total of 268 GIST cases, diagnosed and treated between January 2000 and July 2009, were retrospectively identified from the hospitalization archives of Beijing Cancer Hospital, Beijing, China. One hundred ninety-five of these cases received surgical resection. The inclusion criteria for this study were as follows: (1) primary GIST cases; (2) R0 resection cases without tumor rupture; and (3) no other primary tumors. Using these criteria, 124 cases were identified from surgical records and pathological reports. With the exception of 10 cases out of interval, a total of 114 cases were enrolled in the current study. Follow-up information was obtained from outpatient medical records or from patients and/or their relatives by telephone interview using a follow-up questionnaire.

### Clinicopathological Variables

GIST was diagnosed using an immunohistochemistry panel that included CD117, CD34, DOG-1, desmin, SMA, S-100, and Ki67 and confirmed by positive staining for CD117 and/or DOG-1 with or without desmin (−), SMA (−), and S-100 (−). Clinical data such as gender, age, gastrointestinal bleeding, tumor site, tumor size, and surrounding tissue invasion were obtained from medical records. Gastrointestinal bleeding was confirmed by history of present illness and laboratory test reports revealing the presence of black stools, hematemesis or positive Fecal Occult Blood (FOB), with or without anemia. Surrounding tissue invasion was confirmed via surgical records and pathological reports. Tumor size was measured by pathologists either before or after formalin fixation. The histopathological variables analyzed for each tumor were as follows: mitotic index (number of mitoses per 50 randomly selected high-power microscopic fields [HPFs]), tumor necrosis (presence or absence), predominant cell type (spindle, epithelioid, or mixed), and cellularity (high or moderate-paucicellular). All of the above pathological information of tumors were evaluated by 2 independent pathologists (Li ZW and Dong B), and any differences in interpretation were resolved by consensual agreement.

### Tissue Microarray & IHC Staining

Formalin-fixed, paraffin-embedded tissue block and corresponding H&E-slides were used for tissue TMA sampling. Two pathologists (Li ZW and Dong B) reviewed all the slides and selected the areas for tumor sampling. A tissue arraying instrument (Beecher Instruments, Silver Spring, MD, USA) was used to punch 1.0-mm-diameter cylinders in each donor tissue block and re-embed the same into a corresponding recipient paraffin block. Multiple sections (4-um thick) were cut from the TMA tissue array and prepared for subsequent IHC staining.

TMA slides were deparaffinized and dehydrated through a series of xylene and alcohol solutions. Tissue sections were blocked for peroxidase activity with 3% hydrogen for 10 minutes. Antigen retrieval was performed in EDTA buffer (pH 8.0) by Dako PT Link at 95°C for 15 min. After being blocked with 5% defatted milk, sections were incubated with primary antibodies in an appropriate dilution at 4°C overnight. The primary antibodies and their dilutions are listed in Table 1. Primary antibodies were detected using the Power vision 2-step histostaining reagent (PV-6001, Dako, Glostrup, Denmark). Finally, slides were visualized with 3,3′-diaminobenzedine and counterstained with hematoxylin.

**Table 1:** Primary antibodies for immunohistochemical analysis.

Antibody	Clone	Manufacturer	Dilution
Ki67	MIB-1	Dako, Denmark	1∶100
p16^INK4A^	6H12	ZETA Corporation, Sierra Madre, CA, USA	1∶400
p27^KIP1^	EP104	Epi-Plus™, Novus Biologicals, Littleton, CO, USA	1∶500
p53	DO7	Dako, Denmark	1∶100
SKP2	2CBD9	Invitrogen™, Life Technologies, Carlsbad, CA, USA	1∶200
CD133	AC133	Abnova, Taipei, Taiwan	1∶100
actin	ZMSA-5	ZETA Corporation, Sierra Madre, CA, USA	1∶200

### Evaluation of Biomarker Expression

We estimated p16 immunohistochemical staining based on 10 HPFs as previously described by Schneider-Stock et al. [20]. Nuclei of tumor cells with or without cytoplasmic staining were counted according to a 4-point semiquantitative scale [no staining, 0–10% (0); weak, 11–20% (1); moderate, 21–50% (2); strong, >50% (3)]. A cut-off of 20% positivity in at least 10 HPFs was used for prognostic analysis.

For p27, p53, Ki67, and SKP2, cell nuclei immunostaining was evaluated by counting 10 HPFs and a minimum of 500 cells per slide. Two parameters were assessed for each section for p27 and p53: (a) staining intensity, ranging from 0 to +3 and (b) percentage of positively stained cells, ranging from 0% to 100%. A scoring index was then calculated by multiplying staining intensity by the percentage of positively stained cells [21]. In previous studies, the cut-off point of continuous variables was determined by receiver–operator characteristic (ROC), using recurrence as a basis [22]. However, the postoperative recurrence time varied dramatically in the present study (4 to 108 months), which may reflect differences in the biological behavior of the tumors. Based on both the data features and the ROC curve, p53 was classified into 2 categories, index <10 and index ≥10, and p27 was classified into 3 categories, index <100, index between 100–200, and index >200. Although, different cut-off points for Ki67 have been described, varying from 0.82% to 10%, 3% and 5% have been the cut-offs used most often for GISTs [22], [23]. Here, a 5% cut-off was found to be better than 3% to classify cases using Kaplan-Meier curves. High nuclear SKP2 expression was defined as >10% positive nuclei as previously described by Oliveira et al. in soft-tissue sarcomas [24].

For CD133 and actin, which show cytoplasmic staining, expression was categorized into (−), (+), (++), and (+++), according to the extent and intensity of the staining in true neoplastic tumor cells. Actin expression was negative in most cases; therefore, cases with (+), (++), and (+++) expression were integrated into the “positive expression” category.

### Mutational Analysis

Genomic DNA was extracted from paraffin-embedded tumor specimens using E.Z.N.A. FFPE DNA Kit (Lot. D3399-1, OMEGA, USA), according to the manufacturer’s instructions. DNA fragments that aligned respectively with c-KIT gene exons 9, 11, 13, and 17 and PDGFRα gene exons 12 and 18 were amplified by PCR using different primers (designed using software Primer Premier 5.0). Each PCR reaction system consisted of 2 µL of 10× LA PCR buffer II, 2 µL of 10 mmol/L dNTPs, 0.2 µL of LA Taq (DRR200A, TAKARA), 2 µL of genomic DNA, and 0.5 µL of each primers (10 µmol/L) in a final volume of 20 µL. The cycling conditions were 94°C for 5 min, 40 cycles of 94°C for 30 s, X°C for 30 s and 72°C for 20 s, with a final extension at 72°C for 10 min (Table S2). PCR products were identified by 3% agarose gel electrophoresis and sequenced with an Invitrogen 3730XL genetic analyzer. The sequencing results were analyzed using Chromas software with a signal to noise ratio >98%. Each sample was sequenced at least twice.

### Statistical Analyses

Recurrence or metastasis was considered the most suitable event for statistical analysis because overall survival could be biased by the introduction of IM as a treatment for patients with metastatic GIST. Relapse-free survival (RFS) was calculated from the date of tumor resection to the date of GIST recurrence or to the last follow-up date, if GIST had not recurred. Patients without recurrence or metastasis at the time of data collection and those who died of other causes were regarded as censored data.

Kaplan-Meier curves were used to visualize clinicopathological factors, biomarker expression, and mutational status with respect to RFS data. Log-rank tests were performed to evaluate the statistical significance of associations at the univariate level, and multivariate Cox regression analysis with a backward selection strategy was carried out to explore independent risk factors. Univariate logistic regression was used to evaluate correlations between variables. Spearman correlation coefficient was also calculated when needed.

All statistical analyses were performed using the Statistical Package for the Social Sciences (SPSS) for Windows version 16.0 (SPSS Inc, Chicago, IL), and *P*<0.05 was considered statistically significant.

## Results

### Patient and Tumor Characteristics

114 patients comprised of 67 men and 47 women (age range, 15–82 years; median, 59 years at the time of diagnosis) were included in the present study. Gastrointestinal bleeding existed as a primary symptom in 44 patients. GIST locations included stomach (73 cases), duodenum (5 cases), intestine (24 cases), colon and rectum (7 cases), and intraperitoneum with unknown primary origin (5 cases). GIST resection was performed by open laparotomy, except in 6 patients who underwent laparoscopic resection. Tumors ranged in size from 0.6 to 30 cm (median, 7 cm). Microscopically, the spindle cell type was the most common (88 cases), followed by epithelioid cell type (15 cases) and mixed type (11 cases). The mitotic index per 50 HPFs was less than 5 in 83 GISTs, between 6 and 10 in 20 GISTs, and over 10 in 11 GISTs. More detailed clinicopathological data are shown in Table 2.

**Table 2:** Patient clinicopathological characteristics.

Clinicopathological feature		NO. (%)
Gender	male	67 (59)
	female	47 (41)
Age (years)	<60	72 (50)
	≥60	72 (50)
	median (years)	59
Gastrointestinal bleeding	yes	44 (39)
	no	70 (61)
Primary tumor site	stomach	73 (65)
	duodenum	5 (4)
	intestine	24 (21)
	colorectum	7 (6)
	intraperitoneally with unknown primary origin	5 (4)
Tumor number	single	110 (97)
	multiple	4 (3)
Primary tumor size	>10 cm	31 (27)
	5.1–10 cm	44 (39)
	0–5 cm	39 (34)
Mitotic index (per 50 HPFs)	>10	11 (10)
	6–10	20 (18)
	0–5	83 (72)
Surrounding tissue invasion	yes	24 (21)
	no	90 (79)
Tumor necrosis	yes	67 (59)
	no	47 (41)
Predominant cell type	spindle	88 (77)
	epithelioid	15 (13)
	mixed	11 (10)
Cellularity	high	57 (50)
	moderate	45 (39)
	paucicellular	12 (11)
Postoperative IM treatment	yes	21 (18)
	no	85 (75)
	non-standard*	8 (7)
AFIP-Miettinen criteria	very low risk	30 (26)
	low risk	23 (20)
	moderate risk△	16 (14)
	high risk▴	43 (38)
	insufficient data	2 (2)

* 8 patients stopped IM treatment at mean of 2.2 months due to severe adverse effects;

△ one case with double tumors upstaged from low-risk;

▴ two cases with double tumors upstaged from moderate-risk; IM, imatinib mesylate.

All cases were analyzed for risk stratification according to AFIP-Miettinen criteria. Four cases with double primary GISTs were found in the database, and until now, no consensus has been formed as to whether multiple GISTs should be up-staged. Since multiple origins may be indicative of a more aggressive tumor, these cases were up-staged in the AFIP-Miettinen criteria (low to moderate, 1 case; moderate to high, 1 case; insufficient to high, 1 case), with the exception of cases already in the highest risk category.

Postoperative adjuvant treatment varied among the 114 patients included in this study and was considered during the analysis. Twenty-one (18%) patients received standard adjuvant treatment by imatinib mesylate (400 mg/d from 6 months to 3 years), while 85 patients (75%) had no postoperative adjuvant treatment. Eight patients (7%) stopped imatinib mesylate no longer than 4 months (1 to 4 months; mean, 2.2 months) due to severe adverse effects. These 8 cases were excluded from the univariate analysis of postoperative adjuvant therapy.

### Immunohistochemical Findings

**Figure 1:**
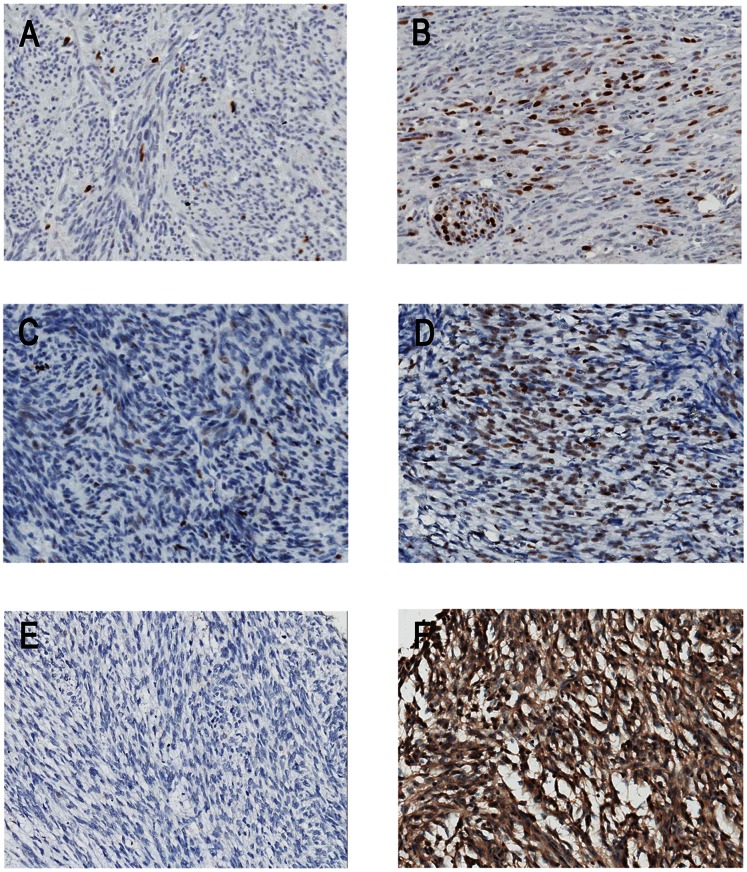
Representative examples of Ki67, p53, and SKP2 expression in GIST. (A–B): Ki67 nuclei staining in 3% (A) and 20% (B) of cells on TMA slide. (C–D): p53 nuclei staining in 20% of cells with moderate intensity (C) and 40% of cells with strong intensity (D) on TMA slide. The index for C and D is 40 (40 = 2*20%*100) and 120 (120 = 3*40%*100), respectively. (E–F): Negative (<10%) (E) and positive (>10%) (F) SKP2 nuclei with cytoplasmic staining on TMA slide (×200).

The complete panel of immunohistochemical markers (Ki67, p16, p27, p53, SKP2, CD133, and actin) was assessed in all cases. For p16, the nuclei stained tumor cells were counted according to the previously described 4-point semiquantitative scale, and a cut-off value of 20% positivity in at least 10 HPF was used for prognostic analysis. p16 nuclei staining was observed in ≤20% of the cells in 59 patients (52%) and in >20% of the cells in 55 patients (48%). Cytoplasmic staining of CD133 was evaluated according to the extent and intensity of the staining and categorized into one of 4 groups: (−), (+), (++), and (+++). Actin expression was considered negative in 74 patients (66%) and positive, which included (+), (++), and (+++), in 40 patients (34%). More detailed data are shown in Table 3. Representative examples of Ki67, p53, and SKP2 staining are displayed in Figure 1.

**Table 3:** Patient clinicopathological characteristics.

Immunohistochemical biomarkers		NO. (%)
Ki67	<5%	81 (71)
	≥5%	33 (29)
p16	≤10%	39 (34)
	11–20%	20 (18)
	21–50%	29 (25)
	>50%	26 (23)
p27 (index*)	<100	27 (24)
	100–200	38 (33)
	>200	49 (43)
p53 (index*)	<10	59 (52)
	≥10	55 (48)
SKP2	<10%	87 (76)
	>10%	27 (24)
CD133	(−)	19 (17)
	(+)	35 (30)
	(++)	29 (25)
	(+++)	31 (28)
actin	(−)	74 (66)
	(+)	23 (20)
	(++)	13 (11)
	(+++)	4 (3)

* Scoring index: staining intensity × percentage of positively stained cells.

### KIT and PDGFRα Mutations

The KIT and PDGFRα mutational hot spots were successfully evaluated in 92 of 114 GISTs (80%). The overall mutation rate was 90% (83 cases), with KIT mutations in 81 (88%) and PDGFRα in 2 (2%) cases. All identified KIT and PDGFRα mutations are listed in Table S3.

The exon that most commonly harbored mutations was the KIT exon 11, in which mutations were observed in 67 cases (72.8%). The mutations found in this gene included 26 deletions (38.8%), 29 point mutations (43.2%), 11 deletion plus point mutations or insertions (16.4%), and 1 duplication (1.5%). Thirty-seven cases (55%) of deletions in KIT exon 11 were found in total. Deletions leading to the loss of codons 557–558 were found in 24 cases (64.8% of all exon 11 deletion cases), making this the most commonly identified deletion in the present study.

Fourteen mutations in KIT were identified in exons other than exon 11. Seven mutations were found in exon 9 leading to A502_Y503 duplication, 5 point mutations in exon 17, leading to N822K or N822R substitution, and 1 point mutation in exon 13, resulting in K642E substitution. In addition, 1 GIST was found in which exon 13 and 17 point mutations were present.

All KIT-WT tumors were examined for PDGFRα mutations. Only 2 mutations in PDGFRα exon 18 were identified, and no mutation was identified in PDGFRα exon 12. Of the 2 mutations found in exon 18, one was a deletion leading to Del 843–846, and the other was a point mutation resulting in D842V.

### Univariate RFS Analysis

Univariate analysis was performed using the clinicopathological parameters, immunohistochemical markers, and gene mutation status previously described. The median follow-up time was 50 months (range, 4 to 150 months) for patients free of recurrence, and the 1, 3, 5-year RFS rate was 88.6%, 71.9%, and 66.3%, respectively. GIST recurred or metastasized after surgery in 42 of the 114 cases during the follow-up. Fifteen of these cases suffered from liver metastasis, while the other 27 cases had recurrence in the abdominal cavity. Univariate analysis revealed that male gender (*P = *0.024), gastrointestinal bleeding (*P = *0.029), tumor size >5 cm (*P*<0.001), non-gastric site (*P*<0.001), mitotic index >5/50HPFs (*P*<0.001), necrosis (*P = *0.003), epithelioid or mixed cell type (*P = *0.030), surrounding tissue invasion (*P = *0.002), AFIP-Miettinen high and moderate risk (*P*<0.001), Ki67≥5% (*P*<0.001), p16>20% (*P = *0.021), p53 index ≥10 (*P = *0.012), SKP2>10% (*P*<0.001), and KIT exon 11 deletion (*P = *0.022) were significantly associated with reduced recurrence free survival (RFS). Detailed data are shown in Table 4.

**Table 4:** Univariate analysis of factors influencing RFS.

Factor		5-year RFS rate (%)	*P* value
Gender	Male: female	55.7∶80.6	0.024
Age	<60: ≥60	71.6∶61.0	NS (0.669)
Gastrointestinal bleeding	Yes: no	50.7∶75.7	0.029
Primary tumor site	Gastric: non-gastric	80.5∶39.7	<0.001
Primary tumor size	≤5 cm: 5.1–10 cm: >10 cm	89.7∶67.5∶36.7	<0.001
Mitotic index (per 50 HPFs)	≤5∶6–10: >10	80.4∶35.0∶18.2	<0.001
Surrounding tissues invasion	Yes: no	40.0∶73.1	0.002
Tumor necrosis	Yes: no	55.0∶82.4	0.003
Predominant cell type	Spindle: epithelioid/mixed	68.8∶57.7	0.03
Cellularity	High: moderate/paucicellular	64.2∶68.7	NS (0.551)
Postoperative IM treatment	Yes: no	79.2∶66.6	NS (0.434)
AFIP-Miettinen criteria	Very low: low: moderate: high	100∶91.7∶66.7∶30.2	<0.001
Ki67	<5%: ≥5%	79.4∶27.4	<0.001
p16	≤20%: >20%	76.4∶54.8	0.021
p27 (index*)	<100∶100–200:>200	65.7∶64.6∶68.6	NS (0.846)
p53 (index*)	<10: ≥10	76.3∶54.9	0.012
SKP2	≤10%: >10%	74.6∶40.0	<0.001
CD133	(−): (+): (++): (+++)	52.6∶68.0∶57.5∶80.4	NS (0.183)
actin	(−): (+, ++, +++)	66.5∶66.3	NS (0.915)
Mutation status	KIT exon 11 deletion:	48.3∶74.3∶72.0	0.022
	KIT exon 11 others: others		

* Scoring index: staining intensity × percentage of positively stained cells; NS, not significant.

### Multivariate RFS Analysis

Only those variables showing a statistically significant relationship with RFS in the univariate analysis were entered in the Cox’s proportional-hazard model. The AFIP-Miettinen stratification scheme was excluded from the analysis since it is based on mitotic index, tumor size, and tumor site and correlated with each of them strongly. Further correlation analysis was performed for the candidate factors before multivariate analysis. It showed that Ki67, SKP2, and p53 correlated with each other strongly (Table 5), and the high expression of each one has a strong correlation with the AFIP-Miettinen high-risk category (Table S4). Based on these findings, 4 models for multivariate analysis were developed, using the same patients and other significant factors found in the univariate analysis. In model A, analysis included Ki67 without SKP2 and p53, while in models B and C, analysis included SKP2 and p53 instead of Ki67, respectively. In model D, Ki67, SKP2, and p53 were all included in the multivariate analysis. Mutation status information was identified successfully in only 92 cases, resulting in the loss of 22 samples in multivariate analysis. This category was set to “missing,” and the relative risk (RR) and *P* value of the “missing” data set was not taken into account.

**Table 5:** Correlation analysis between Ki67, SKP2, and p53.

Variable		Ki67	SKP2	p53
p53	Correlation coefficient	0.390	0.412	1.000
	Sig.(2-tailed)	<0.001	<0.001	
SKP2	Correlation coefficient	0.418	1.000	
	Sig.(2-tailed)	<0.001		
Ki67	Correlation coefficient	1.000		
	Sig.(2-tailed)			

The 4 models developed for multivariate analysis yielded different results. In model A, high Ki67 expression and gastrointestinal (GI) bleeding were statistically significant indicators of poor RFS (RR = 2.43, 95% CI: 1.18–4.99 and RR = 2.29, 95% CI: 1.18–4.47, respectively). In contrast, highSKP2 expression was a strong significant indicator in model B (RR = 2.91, 95% CI: 1.41–5.99), and GI bleeding was also valuable with near statistical significance (RR = 1.88, 95% CI: 0.98–3.64). In model C, GI bleeding was again a significant indicator of poor RFS (RR = 2.31, 95% CI: 1.18–4.51), while p53 did not show any significant influence. Importantly, in model D which included Ki67, SKP2, and p53, only high SKP2 expression was an independent risk factor (RR = 2.91, 95% CI: 1.41–5.99, *P = *0.004), and GI bleeding was a potentially valuable factor with a *P* value nearing the significance threshold (RR = 1.88, 95% CI: 0.98–3.64, *P = *0.059).

Mutation status also showed a statistically significant influence on RFS. When compared to non-KIT exon 11 mutations, KIT exon 11 deletions were indicative of poor RFS in every model (RR = 2.73, 95% CI: 1.04–7.16, *P = *0.041, model D). In addition, tumor site, tumor size, and mitotic index were also stable independent risk factors in every model (Table 6). Since these results may have been biased by postoperative adjuvant IM therapy, the previous analyses were repeated in stratified non-adjuvant therapy group patients, which provided similar results, except that KIT exon 11 deletions did not significantly influence RFS in these patients (Table S5).

**Table 6:** Multivariate analysis of factors influencing RFS.

	Model A	Model B	Model C	Model D	
	RR (95% CI)	RR (95% CI)	RR (95% CI)	RR (95% CI)	*P* value
GI bleeding (no*: yes)	2.29 (1.18–4.47)	1.88 (0.98–3.64)	2.31 (1.18–4.51)	1.88 (0.98–3.64)	0.059
Site (gastric*: non-gastric)	4.30 (2.12–8.75)	5.52 (2.60–11.69)	6.96 (3.10–15.59)	5.52 (2.60–11.69)	<0.001
Size (≤5 cm*: 6–10 cm)	1.54 (0.49–4.89)	1.34 (0.41–4.38)	2.46 (0.72–8.32)	1.34 (0.41–4.38)	0.624
(≤5 cm*: >10 cm)	3.53 (1.16–10.70)	3.51 (1.15–11.73)	5.65 (1.81–17.70)	3.51 (1.15–11.73)	0.027
Mitotic index (0–5*:5–10/50HPFs)	5.15 (2.40–11.09)	7.00 (3.02–11.87)	6.27 (2.66–14.80)	7.00 (3.02–11.87)	<0.001
Ki67 (<5%*: ≥5%)	2.43 (1.18–4.99)			1.38 (0.59–3.24)	0.462
SKP2 (<10%*: >10%)		2.91 (1.41–5.99)		2.91 (1.41–5.99)	0.004
p53 (index <10*: index ≥10)			1.10 (0.47–2.58)	0.65 (0.24–1.78)	0.403
Mutation					
(non-exon 11*: exon 11 others)	1.47 (0.48–4.53)	2.23 (0.66–7.48)	1.43 (0.45–4.54)	2.23 (0.66–7.48)	0.195
(non-exon 11*: exon 11 deletion)	2.83 (1.08–7.43)	2.73 (1.04–7.16)	3.39 (1.29–8.88)	2.73 (1.04–7.16)	0.041

Model A includes analysis of Ki67 without SKP2 and p53; model B includes SKP2 without Ki67 and p53; model C includes p53 without Ki67 and SKP2, and model D includes Ki67, SKP2, and p53.

* represents reference. GI, gastrointestinal; RR, relative risk; CI, confidence interval.

### Clinical Benefits

To validate the potential prognostic value of these new factors, we compared statistic models including the conventional prognostic factors before and after the addition of the novel factors elucidated in the present study. We used the −2 times log likelihood ratio (−2*log l) to evaluate the goodness of fit. The smaller the −2*log l value, the better was the goodness of fit. The −2*log l value of the model with traditional 3 factors was 253.812; when “GI bleeding” was incorporated, the value became 249.297; when “SKP2 high expression” was incorporated, it became 245.274; when “KIT exon 11 deletion” was incorporated, it became 249.894; when all of 3 new factors were incorporated, it became 239.587. These results revealed that when each of the new factors was incorporated into the conventional model, the goodness of fit improved.

**Figure 2:**
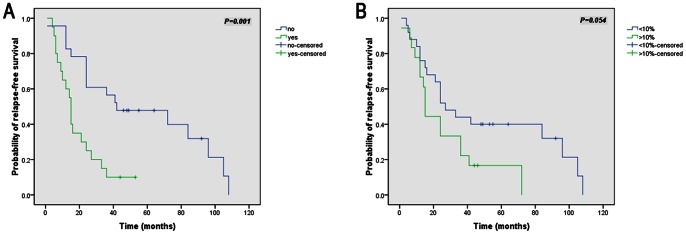
Relapse-free survival analysis of 43 patients in the AFIP-Miettinen criteria high-risk category. (A–B): Kaplan-Meier curve analysis illustrating a worse relapse-free survival for AFIP high-risk patients with gastrointestinal bleeding (A) and with SKP2 high expression (B).

In addition, to further explore the clinical benefits of including these predictors in current risk stratification systems, we created 4 individual subgroups based on the AFIP-Miettinen criteria (very low, low, moderate, and high risk) and the new factors from this study were utilized to further discriminate the patients in each subgroup. Our results revealed GI bleeding to be significantly associated with a further reduction of the RFS in the high-risk category (*P = *0.001, Figure 2A). Moreover, SKP2>10% also showed a potential trend for poor prognosis with *P* = 0.054 (Figure 2B) in the high-risk category. In contrast, no obvious differences were observed in other 3 subgroups by inclusion of the new factors.

**Figure 3:**
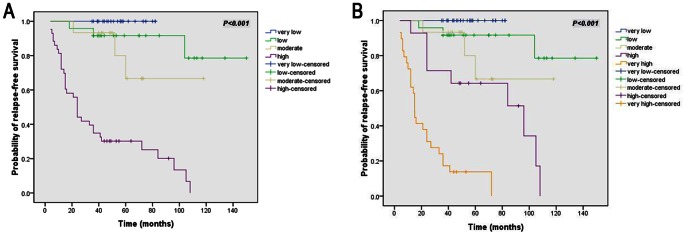
Relapse-free survival analysis of 114 GIST patients. (A–B): Kaplan-Meier curve analysis illustrating a better risk stratifying ability for “modified AFIP criteria” (B) than AFIP-Miettinen criteria (A).

Based on these results, we consider that the current AFIP-Miettinen criteria could be further improved as the “modified AFIP criteria”. In such an improved classification, the very low, low, and moderate risk categories would remain consistent with those in the original criteria; however, the previous high-risk category could be further subdivided into the “high risk” and “very high risk” groups. For example, a patient from the high-risk category according to the AFIP-Miettinen criteria shows the presence of either GI bleeding or SKP2>10%, he/she would be classified into the “very high risk” group in the “modified AFIP criteria”, while if both factors are negative, he/she would be classified as “high risk”. The survival curves showed that the “modified AFIP criteria” may have a better ability to stratify postoperative primary GIST patients (Figure 3). And this modified stratification for high-risk postoperative primary GIST patients can enable to improved selection of appropriate adjuvant therapy.

## Discussion

Here, our univariate analysis showed that clinicopathological parameters such as male gender, tumor necrosis, epithelioid cell type, and surrounding tissue invasion were significant predictors of a poor prognosis in GISTs. Our results are generally in agreement with other studies showing that, in addition to high cellularity–which was not observed in our study–these parameters were poor prognostic indicators on univariate and multivariate analyses [9]–[11]. Tumor necrosis, an important prognostic factor in some sarcomas [25], appeared to correlate with tumor size, surrounding tissue invasion, and Ki67 expression in our primary GIST patients. In addition, surrounding tissue invasion, a symbol of T4 and poor prognosis in most malignant tumors, occurred more readily in larger tumors, intestinal tumors, and necrotic tumors. However, the only clinicopathological factor that showed significant correlation with RFS in our multivariate analysis was gastrointestinal bleeding.

Gastrointestinal bleeding is one of the most common GIST symptoms, appearing in approximately 40% patients, although previous studies have rarely described its influence on RFS. Our data showed that GI bleeding was a risk factor in both univariate analysis and multivariate analysis models A and C. Although the *P*-value failed to reach statistical significance in models B and D, GI bleeding still showed obvious relative risk. In addition, no obvious correlation was observed between GI bleeding and other factors, except tumor necrosis. Kaplan-Meier curves validated the further stratification value of GI bleeding in AFIP-Miettinen high-risk patients, implying that GI bleeding might be a potential parameter worthy of further study. Although GI bleeding is a sign of mucosal invasion, which has been examined previously [26], its significant association with RFS is hard to explain based on this reason alone; we consider that this association may be due to hypoimmunity caused by anemia and/or a low KPS score.

GIST clinical behaviors have also been associated with the expression of several immunohistochemical biomarkers, although their prognostic value in GIST remains controversial. For example, Ki67, a nuclear protein associated with cellular proliferation, has been associated with poor outcomes in GIST patients [9], [23], [24], while others have reported contradictory or inconclusive findings with Ki67 labeling [27], [28]. Our results showed that high nuclei Ki67expression correlated with necrosis and high mitotic index, which also reflect cellular proliferation. Interestingly, high nuclei Ki67expression also strongly correlated with high SKP2 and p53 expression. In the multivariate analysis model A, Ki67 was an independent risk factor for poor RFS. However, when SKP2 and p53 were also included in regression model D, Ki67 was replaced by SKP2. A correlation between Ki67 and SKP2 has been found in several tumor types, including GIST [10], [25], [29].

SKP2 promotes the degradation of phosphorylated cyclin-dependent kinase inhibitor p27^ Kip1^ [30], and SKP2 overexpression has been shown to contribute to p27^Kip1^ degradation in lymphomas [31], colorectal cancer [32], and GIST [10]. Similar to another report [29], this inverse correlation was not observed in the current study; however, SKP2 was observed to positively correlate with Ki67 and p53 levels. Additionally, Di Vizio et al. reported that SKP2 expression correlates with several parameters of GIST malignant potential and suggested that SKP2 might play an important role in predicting the aggressive potential of GIST [10], as it does in soft-tissue sarcomas [24], [33]. To the best of our knowledge, the significance of SKP2 overexpression as an independent prognostic marker in GIST has not been previously reported. Here, SKP2 not only associated with the mitotic index, high-risk category, and Ki67 and p53 expression, but also was an independent negative prognostic factor for RFS in multivariate analysis models B (with SKP2, without Ki67 and p53) and D (with all of SKP2, Ki67, and p53). These findings implicate SKP2 as an important biomarker in predicting the biological behaviors of GISTs and is worthy of further investigation.

The prognostic significance of the cell-cycle regulatory proteins (CCRPs) p16, p27, and p53 in GIST is still under debate. Constitutive KIT/PDGFR activation promotes proliferation and inhibits apoptosis of neoplastic cells through the CCRP signaling pathway, which is negatively regulated by p16 and p27, while p53 acts as a cross-talk regulator among CCRPs [34]. Although reports have been conflicting [21], [35], [36], decrease or loss of p16 and p27 expression can be a predictor of poor prognosis [20], [37], whereas p53 overexpression appears to correlate with increased malignant risk in GIST in some studies [28], [38]. Here, p16, p27, and p53 did not have a significant influence on RFS in multivariate analysis. However, high p16 and p53 expression was found to be an unfavorable factor in univariate analysis, and p53 overexpression correlated with several parameters of malignant potential (mitotic index, AFIP high risk, cell type, Ki67 and SKP2 expression), indicating an important auxiliary role for p53 in predicting the aggressive potential of GIST.

A recent study reported that CD133 is highly expressed in a subset of predominantly gastric GISTs with KIT exon 11 mutations and poor prognosis [12]. In addition, another study reported that negative actin expression may act as a poor prognostic factor [13]. Here, high CD133 expression indeed correlated with gastric location and KIT exon 11 mutations; however, no obvious correlation between CD133 and actin expression with RFS in either univariate or multivariate analysis was observed.

The importance of KIT and PDGFRα mutations as prognostic indicators remains controversial, although their predictive value on tyrosine kinase inhibitor response is now clearer. Some large-scale studies have shown that KIT gene deletions indicate a high metastatic risk and poor prognosis [16]. Conversely, tumors with point mutations or duplications in the distal part of KIT exon 11 are less aggressive than those with deletions [17]. Moreover, patients with KIT exon 11 deletions involving codons 557 and/or 558 may have a less favorable outcome than patients with no or different KIT mutations [39]. Here, KIT and PDGFRα mutations were found in 88% and 2% of cases, respectively, similar to mutation frequencies found in some studies [39], [40], but higher than others [16], [41]. These findings imply that KIT exon 11 deletions may be a better indicator of aggressiveness in GISTs. However, in the present study, when cases were categorized into “KIT exon 11 mutations vs. others” or “KIT exon 11 557–558 deletions vs. other KIT exon 11 deletions vs. others,” no significant differences were observed between groups.

In multivariate analysis, KIT exon 11 deletions were observed to be an independent risk factor compared to non-exon 11 cases. This finding suggests that non-exon 11 cases should be considered less aggressive, while KIT exon 11 deletions could be an indicator of poor RFS. However, the prognostic value of non-KIT exon 11 cases may vary dramatically according to the location and type of the mutation [18], [19].

Our study has certain limitations. Care must be taken when interpreting our results given the limited sample size, large proportion of censored cases, and the somewhat subjective nature of the histopathological evaluation and biomarker cut-off value selection, which may impact the accuracy of the results. Moreover, mutation status information was not identified successfully in all cases due to difficulties in DNA extraction from older specimens, which may have influenced the evaluation of the mutation status with regard to RFS. Given the uncertainties and the limitations of this study, another study using larger patient samples with longer follow-ups is needed to clarify these issues.

In summary, our findings demonstrate that gastrointestinal bleeding, SKP2 high expression, and KIT exon 11 deletions may be useful indicators of poor RFS in primary GIST patients. Gastrointestinal bleeding and SKP2 high expression showed a good ability to stratify high-risk patients further, and we consider that AFIP-Miettinen criteria may be improved by including these predictors, particularly for the high-risk patient category.

## Supporting Information

Table S1 Definition of the risk categories in AFIP-Miettinen criteria. HPF, high-power field.
(DOCX)

Table S2 Primers, anneal temperature (Tm) and product size of each exon.
(DOCX)

Table S3 KIT and PDGFRA mutations identified in this study.
(DOCX)

Table S4 Correlation analysis between AFIP-Miettinen criteria and biomarkers.
(DOCX)

Table S5 Multivariate analysis of factors inﬂuencing RFS in stratified non-adjuvant therapy group patients. Model A includes analysis of Ki67 without SKP2 and p53; model B includes SKP2 without Ki67 and p53; model C includes p53 without Ki67 and SKP2, and model D includes Ki67, SKP2, and p53. *represents reference. GI, gastrointestinal; RR, relative risk; CI, confidence interval.
(DOCX)
