# Supplementary material for: SKP2 High Expression, KIT Exon 11 Deletions, and Gastrointestinal Bleeding as Predictors of Poor Prognosis in Primary Gastrointestinal Stromal Tumors
Source: PLoS One. 2013 May 17;8(5):e62951. doi: 10.1371/journal.pone.0062951 (PMC3656858; doi:10.1371/journal.pone.0062951)
Supplement: Table S3 — KIT and PDGFRA mutations identified in this study. (DOCX) [file pone.0062951.s003.docx]

**Table S3:** KIT and PDGFRA mutations identified in this study
