# Supplementary material for: SKP2 High Expression, KIT Exon 11 Deletions, and Gastrointestinal Bleeding as Predictors of Poor Prognosis in Primary Gastrointestinal Stromal Tumors
Source: PLoS One. 2013 May 17;8(5):e62951. doi: 10.1371/journal.pone.0062951 (PMC3656858; doi:10.1371/journal.pone.0062951)
Supplement: Table S4 — Correlation analysis between AFIP-Miettinen criteria and biomarkers. (DOCX) [file pone.0062951.s004.docx]

**Table S4:** Correlation between AFIP-Miettinen criteria and biomarkers
