# Supplementary material for: SKP2 High Expression, KIT Exon 11 Deletions, and Gastrointestinal Bleeding as Predictors of Poor Prognosis in Primary Gastrointestinal Stromal Tumors
Source: PLoS One. 2013 May 17;8(5):e62951. doi: 10.1371/journal.pone.0062951 (PMC3656858; doi:10.1371/journal.pone.0062951)
Supplement: Table S5 — Multivariate analysis of factors inﬂuencing RFS in stratified non-adjuvant therapy group patients. Model A includes analysis of Ki67 without SKP2 and p53; model B includes SKP2 without Ki67 and p53; model C includes p53 without Ki67 and SKP2, and model D includes Ki67, SKP2, and p53. *represents reference. GI, gastrointestinal; RR, relative risk; CI, confidence interval. (DOCX) [file pone.0062951.s005.docx]

**Table S5:** Multivariate analysis of factors inﬂuencing RFS in stratified non-adjuvant therapy group patients

Model A includes analysis of Ki67 without SKP2 and p53; model B includes SKP2 without Ki67 and p53; model C includes p53 without Ki67 and SKP2, and model D includes Ki67, SKP2, and p53. *represents reference. GI, gastrointestinal; RR, relative risk; CI, confidence interval
